# Total femur replacement in a patient with chronic persistence osteomyelitis – A case report

**DOI:** 10.1016/j.ijscr.2021.106067

**Published:** 2021-06-09

**Authors:** Dung Tran Trung, Hieu Nguyen Dinh, Ngoc Thanh Le, Long Hoang Luong, Tran Thuy Nguyen

**Affiliations:** a Hanoi Medical University, Hanoi, Viet Nam; b E Hospital, Hanoi, Viet Nam; c University of Medicine and Pharmacy, Vietnam National University, Hanoi, Viet Nam

**Keywords:** Chronic persistent osteomyelitis, Total femur replacement, Case report

## Abstract

**Introduction and importance:**

Total femoral replacement (TFR) is a salvage surgical procedure that has been indicated mainly for oncologic indication to avoid lower limb amputation but has recently been indicated for non-oncological disorders.

**Case presentation:**

We report the case of a 63-year-old male with chronic osteomyelitis of the left femur, severe pain and bone deformation, the risk of amputation in this patient was very high. The patient underwent total femur replacement (TFR) with a modular mega-prosthesis. TFR was conducted in two phases. The first one consists of femur resection followed by placement of antibiotic cement; and the second operation was performed after 7 weeks, in which a modular mega-prosthesis was implanted. After a 2-month rehabilitation period, the patient recovered basic ambulation without any complaint of pain or detectable residual infection. The 1-year follow-up was uneventful, with no residual pain or infection. The patient retains normal ambulation and daily function.

**Clinical discussion:**

Chronic persistent osteomyelitis is a hard to manage non-neoplastic disorder that leads to amputation in severe cases. In such patients, TFR would be considered as a salvage therapy that could preserve the patient's anatomical integrity and ambulation.

**Conclusion:**

To the best of our knowledge, this is the first case of TFR for treatment of chronic persistent osteomyelitis in Vietnam. While TFR are still mainly indicated for oncology patients, TFR is anticipated to be performed more frequently for non-oncological disorders where there are extensive femoral bone loss and risk of amputation.

## Highlights

• Chronic persistent osteomyelitis present a high risk of amputation and severse loss of quality of life
• Total femoral replacement (TFR) is a salvage surgical procedure indicated for oncologic disorder to retain normal ambulation.
• This is the first reported case of TFR for Chronic persistent osteomyelitis in Vietnam and one of the few cases worldwide.
• The patient retained normal anatomy, regained basic daily life function and good quality of life.
• TRF is anticipated to be performed more frequently for non-oncological disorders to reduce risk of amputation.

## 1 Introduction

Total femoral replacement (TFR) is a salvage surgical procedure that has been indicated mainly for oncologic indication to avoid lower limb amputation [1]. Since its initial conduction in the mid-20th century, indications have been expanded for other non-oncologic conditions such as congenital bone disorders and metabolic bone diseases, and more rarely osteomyelitis [2]. Total femoral replacement has several advantages in the preservation of femur integrity and allows patients to rehabilitate and regain mobilization earlier. However, most patient with non-neoplastic disorders that require TFR usually undergone several prior arthroplasties and suffer from complications such as failed implants, periprosthetic fractures and infections [3]. It is speculated that TFR will be performed at an increasing rate as indication expand and conduction of arthroplasty rise worldwide [4].

Chronic persistent osteomyelitis is characterized by bone destruction and bone necrosis [5]. Treatment of osteomyelitis usually combines antibiotics with radical surgery of all infection sites including bone and surrounding soft-tissue. However, in case of severe infection, history of recurrence and antibiotic resistance, when other interventions fail, major limb amputation is the last option considered. In such patients, TFR would be considered as a salvage therapy that could preserve the patient's anatomical integrity and ambulation [2]. This case report highlights a patient with 30-years chronic persistent osteomyelitis with extended bone structure damage that requires TFR as the first surgical intervention. In this case, the decision making, patient preparation, procedures and rehabilitation are also discussed in depth. This paper is reported in accordance to the SCARE 2020 guideline [6].

## 2 Case report

A 63 years old male patient came to our attention with persistent, severe pain and loss of ambulation. The patient undergone multiple surgical curettage and radical debridement of the left femur osteomyelitis in the past, he has no history of any systemic diseases or immune disorders. In the last 6 months, the patient complained of frequent intense pain, and having to take high dose pain-killer. This patient was frequently upset by the persistent dull thigh pain that causes a lot of difficulties in daily activity, he is unable to stand and walk upright. Upon further examination we found the patient were apyretic, the left leg was 11 cm shorter compared to the right, gluteus muscle atrophy. Range of motion was measured by standard universal goniometers, follow the neutral-zero measuring method and SFTR reporting system [7]. Active and passive ROM of left hip: flexion 10^0^, extension 0^0^, abduction 0^0^, adduction 0^0^, internal-rotation 0^0^, external rotation 0^0^; Rom of left knee: flexion 10^0^, internal rotation 0^0^, external rotation 0^0^.

**Fig. 1:**
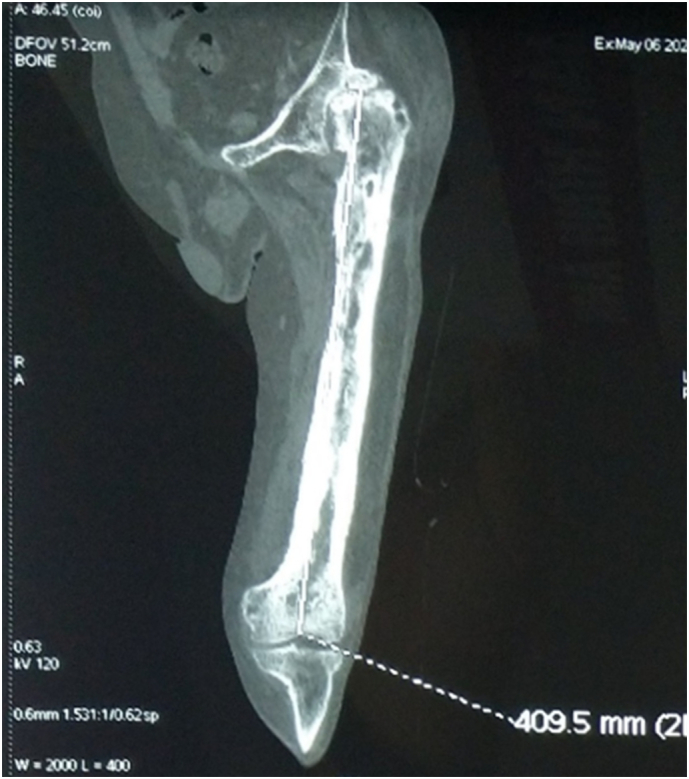
Imaging X-ray and CT-Scanner prior to operation.

Diagnostic imaging with X-ray, CT scanner and MRI revealed a Cierny-Mader Type IV Chronic Osteomyelitis of left femur with degeneration of the left knee and hip joints, Femoroacetabular impingement, communicated acetabular, avascular necrosis of left femoral head (Fig. 1). White blood cell (WBC) 18,700 G/L, erythrocyte sedimentation rate (ESR) 1 h: 76 mm; 2 h: 91 mm; C-reactive protein (CRP): 68.51 mg/L; Pro-calcitonin 1.35 pg/mL. Microbiological test result from bone biopsy confirmed the presence of the *Staphylococcus aureus* causative of the osteomyelitis. After thorough work-up, the definitive diagnosis for this patient was chronic persistent osteomyelitis of the left femur.

With the patient's condition gradually worsen, amputation would be required. Total femoral replacement was indicated as a salvage therapy to preserve anatomy and functionality. We made sure to openly discuss with the patient about all the aspects of the procedure before gaining his consent to proceed with the procedures.

### 2.1 Surgical procedure

The surgical procedure is performed at National E Hospital, Vietnam; the second largest hospital in Hanoi, Vietnam with modern operating theater which regularly perform over 10,000 surgery annually. The lead surgeon is DT Trung, MD., PhD, who is a leading orthopaedician in Vietnam with 20 years of experience and over 20 peer-reviewed papers in Orthopaedic.

**Fig. 2:**
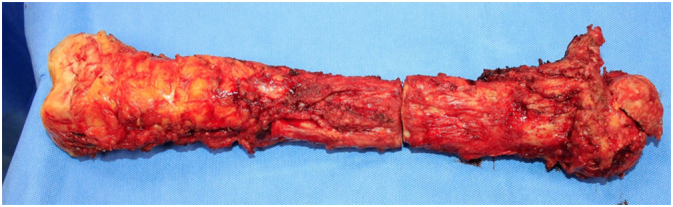
Left femur after resection presented the severity of bone damage and infection.

A thorough and careful evaluation of the patient condition was carried out; the patient was scheduled for a two-stage procedure strategy. *First stage of the procedure:* Under general anesthesia, in supine position, with a lateral incision from 4 cm proximal great trochanter to the antero-lateral of tibia tuberosity, we performed radical surgical debridement of soft tissue and total left femur resection (Fig. 2), antibiotic spacer with biological cement containing vancomycin and gentamycin shaped around a Kuntscher nail was fitted in place of the femur. After surgery, according the antibiotics assay, the antibiotic treatment of *S. aureus* with: intravenous *vancomycin* (after replacing with oral *clindamycin*), *cephalosporin III*, *trimethoprim-cotrimoxazole* (after replacing with *quinolones III*). Antibiotic treatment was continued for 07 weeks after the first operation. At the end of this period the hematological and biochemical test result of the patient was WBC 7000 G/L; ESR 1 h: 26 mm, 2 h: 38 mm; CRP: 27.7 mg/L; Pro-calcitonin: 0.7 pg/dL.

**Fig. 3:**
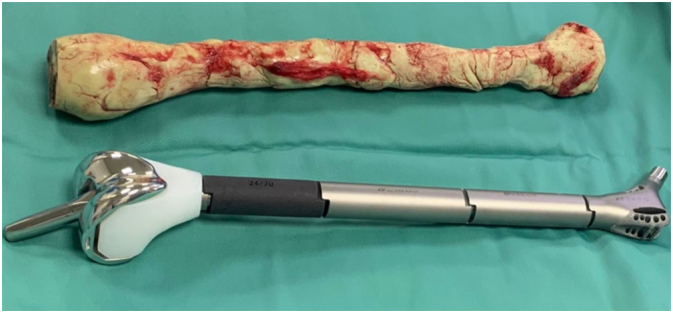
Spacer and Modular mega-prosthesis for Total femur replacement.

*Second stage of the surgical procedure:* Under general anesthesia, follow the incision of the first operation, we removed the spacer, samples were taken for microbial culturing. A femoral modular mega-prosthesis (Beijing Chunlizhengda Medical Instruments Co., Ltd.) (Fig. 3) joined to constraint acetabular 3D-printing (total hip replacement) and a tibial component via a rotating hinge mechanism (total knee replacement) was positioned and secured in place of the femur, after confirming the range of motion of the prosthesis, the surrounding muscle, tendon and soft tissue was sutured and reconstructed anatomically.

### 2.2 Post-operation rehabilitation and follow up

**Fig. 4:**
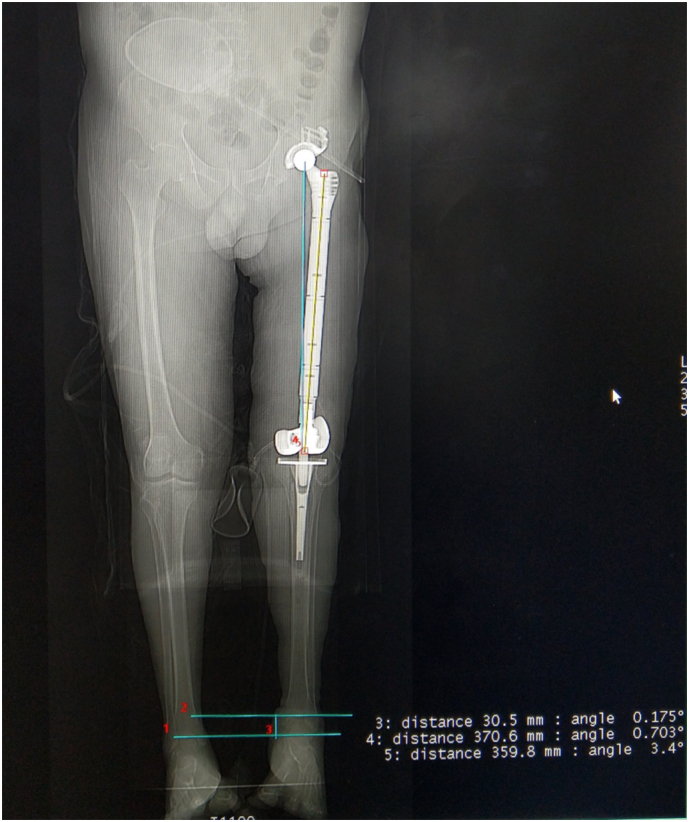
Postoperative X-ray image of the patient presented good structural integrity and anatomical resemblance with a <30 mm length discrepancy between the left and right leg.

After the operation, lower limb discrepancy was reduced from 11 cm to 3 cm, the patient continued three intravenous antibiotics: Vancomycin, Clindamycin, Cephalosporin III. Microbial culture of the samples returned negative for *S. aureus*. Two weeks after operation the active ROM of hip was: 0–80^0^/20^0^–15^0^/15^0^–15^0^; active ROM of knee: 0–40^0^; we also evaluated the patient's satisfaction with the adapted version of the Short Assessment of Patient Satisfaction (SAPS) scale in which the score was 26/28 [8]; Visual analogue scale scoring (VAS) reduced to 1. Postoperative X-ray showed good structural integrity and anatomical closeness (Fig. 4). Rehabilitation was initiated promptly after the procedure which focuses on pain management, maximizing active ROM, strengthening exercises and independent daily life activities. After a 2-month rehabilitation period, the patient recovered basic ambulation without any complaint of pain or detectable residual infection. The 1-year follow-up confirm no pain or any residual infection, the patient retain normal ambulation and daily function, normal imaging. The patient expressed gratitude and was highly satisfied with the result of the treatment and rehabilitation process.

## 3 Discussion

Chronic osteomyelitis is one of the non-malignant bone diseases that pose many challenges in treatment. Management of osteomyelitis depends on the disease staging, but as a generally followed a multi-method approach: the radical surgical intervention in combination with systemic antibiotics [5]. As observed on the pre-operative X-ray of the patient, the whole femur from head of femoral to condylar was a necrotic zone. These bone damage is caused by inflammatory cytokines such as IL-1 or TNF8, the surface of the necrotic bones will be covered with a biofilm, leading to ineffective antibiotic treatment [9]. In cases of severe infection, TFR is a possible solution to eliminate infection and preserve lower-limb function without amputation. To our knowledge there are very few study that report on TFR for the treatment of non-oncologic diseases (4 study in which two were case reports) [2,10,11].

In reviewing of the medical literature, the first surgeon who performed total femur replacement was Buchman in 1965 for Paget, a non-malignant disease [12]. From then until 2015, there were a total of about 200 articles in the 3 main scientific repositories, 65 articles on Pubmed, 75 articles on Embase and 76 articles on Scopus about TFR. The majority of TFR case was indicated for patients with neoplastic diseases [1]. Normally, partial or total femoral replacement surgery is indicated for oncological patients where the lesion could affect the normal function of the lower-limb. Amputation of the affected lower-limb is often indicated for oncological patients, therefor TFR offer an alternative and salvage treatment that preserve the anatomical integrity and ambulation for the patient [13]. A study by Pablo in 2018 reported patients who underwent partial or total femoral replacement for Osteomyelitis, in which three case were TFR [11]. Until recent years in Greece, and the U.S. there are only 2 case reports of TFR for patient with chronic persistent osteomyelitis in 2019. In Vietnam, the first TFR case was performed on patient with osteosarcoma in 2020 [13].

Major bone destruction of the femur accompanied by severe deformation of the acetabular leads to many difficulties in surgery and the risk of hip dislocation after surgery increases significantly. According to the study of Kousterimpas that follow postoperative patient, a liner constraint design helps to prevent dislocation [2]. The hip joint was designed according to the PSI (patient-specific instrument) to prevent dislocation, a hinge-shaped artificial knee joints was added to make up for the loss of surrounding ligament. The design was based on the patient anatomical indexes which are accurately measured on the CT scanner with constrained liner [14].

Postoperative infection is also a major problem for patients who have TFR with accompanied infections. According to a Ramappa 2010 report, when TFR to treat infection around an prosthesis, 1 in 6 patients had re-infection after 3 months of surgery [15]. About 17.2% of patients still had postoperative infections as reported by Corona in 2018. To manage postoperative infections according to many study, the time to use prophylaxis intravenous antibiotics should be 4–6 weeks post-operation, up to 6–8 weeks in some study, followed by oral antibiotics [16].

TFR for a patient with a chronic osteomyelitis is a huge challenge for the surgeons. With patients in the report, the results were promising with a marked improvement in VAS score, patient satisfaction with surgery (SAPS scale). However, it is necessary to continue monitoring and thoroughly prevent any recurrence infection.

## 4 Conclusion

This is the first case of TFR for the treatment of chronic persistent osteomyelitis in Vietnam. While TFR are still mainly indicated for oncology patients, TFR is anticipated to be performed more frequently for non-oncological disorders where there are extensive femoral bone loss and risk of amputation.

## Ethical approval

The study design was reviewed and approved by the Ethical board of National E Hospital. The study complies with the Declaration of Helsinki regarding the use of human samples and identifiable information.

## Sources of funding

None declared.

## CRediT authorship contribution statement

Author DT Trung, HN Dinh, NT Le, LH Luong, TT Nguyen involved in the diagnostic workup, surgery and clinical management of this patient, gather and organize test results, clinical imagings and finalizing the results. Author DT Trung, HN Dinh, NT Le, LH Luong, TT Nguyen contributed in drafting of the manuscript. All authors have read and approved of the final version for publication.

## Guarantor

**MD. PhD.** Tran Thuy Nguyen

ORCID: 0000-0001-7129-0525

## Research registration (for case reports detailing a new surgical technique or new equipment/technology)

Not applicable.

## Consent

Patient's written informed consent was obtained for the study and publication of clinical information. No identifiable information was included in any form.

## Provenance and peer review

Not commissioned, externally peer-reviewed.

## Declaration of competing interest

The authors declared no conflict of interest.

## Appendix A Supplementary data

Video S1 Patient regained normal ambulation and daily life activity after the rehabilitation period. He reported no pain and improved quality of life. Video S1

## References

[1] Ramanathan D., Siqueira M.B., Klika A.K., Higuera C.A., Barsoum W.K., Joyce M.J. (2015). Current concepts in total femoral replacement. World J. Orthop..

[2] Koutserimpas C., Raptis K., Mari A., Kotsirakis A. (2019). Modular megaprosthesis as definite treatment of femur osteomyelitis. G. Chir..

[3] Lentino J.R. (2003). Prosthetic joint infections: bane of orthopedists, challenge for infectious disease specialists. Clin. Infect. Dis..

[4] Berend K.R., Lombardi A.V., Mallory T.H., Adams J.B., Dodds K.L. (2004). Total femoral arthroplasty for salvage of end-stage prosthetic disease. Clin. Orthop. Relat. Res..

[5] Schmitt S.K. (2017). Osteomyelitis. Infect. Dis. Clin. N. Am..

[6] Agha R.A., Franchi T., Sohrabi C., Mathew G., Kerwan A., Thoma A., Beamish A.J., Noureldin A., Rao A., Vasudevan B., Challacombe B., Perakath B., Kirshtein B., Ekser B., Pramesh C.S., Laskin D.M., Machado-Aranda D., Miguel D., Pagano D., Millham F.H., Roy G., Kadioglu H., Nixon I.J., Mukhejree I., McCaul J.A., Chi-Yong Ngu J., Albrecht J., Rivas J.G., Raveendran K., Derbyshire L., Ather M.H., Thorat M.A., Valmasoni M., Bashashati M., Chalkoo M., Teo N.Z., Raison N., Muensterer O.J., Bradley P.J., Goel P., Pai P.S., Afifi R.Y., Rosin R.D., Coppola R., Klappenbach R., Wynn R., De Wilde R.L., Surani S., Giordano S., Massarut S., Raja S.G., Basu S., Enam S.A., Manning T.G., Cross T., Karanth V.K.L., Kasivisvanathan V., Mei Z. (2020). The SCARE 2020 guideline: updating consensus Surgical CAse REport (SCARE) guidelines. Int. J. Surg..

[7] Gerhardt J.J., Rondinelli R.D. (2001). Goniometric techniques for range-of-motion assessment. Phys. Med. Rehabil. Clin. N. Am..

[8] Hawthorne G., Sansoni J., Hayes L., Marosszeky N., Sansoni E. (2014). Measuring patient satisfaction with health care treatment using the Short Assessment of Patient Satisfaction measure delivered superior and robust satisfaction estimates. J. Clin. Epidemiol..

[9] Lüthje F.L., Jensen L.K., Jensen H.E., Skovgaard K. (2020). The inflammatory response to bone infection – a review based on animal models and human patients. APMIS.

[10] Bickels J., Meller I., Henshaw R., Malawer M., Malawer M., Sugarbaker P.H. (2001). Proximal and total femur resection with endoprosthetic reconstruction. Musculoskeletal Cancer Surgery: Treatment of Sarcomas and Allied Diseases.

[11] Corona P.S., Vicente M., Lalanza M., Amat C., Carrera L. (2018). Use of modular megaprosthesis in managing chronic end-stage periprosthetic hip and knee infections: is there an increase in relapse rate?. Eur. J. Orthop. Surg. Traumatol..

[12] B. J (1965). Total femur and knee joint replacement with a vitallium endoprosthesis. Bull. Hosp. Joint Dis..

[13] Trung D.T., Sang N.T.Q., Anh H.T., Thanh T.D., Sam H.M., Hieu P.T., Nam L.V., Tung P.S. (2020). Replacing the entire femur for the cancer treatment of the lower femur: reporting one clinical case. J. Orthop. Trauma Surg. Relat. Res..

[14] Su E.P., Pellicci P.M. (2004). The role of constrained liners in total hip arthroplasty. Clin. Orthop. Relat. Res..

[15] Ramappa M., McMurtry I., Port A. (2010). Direct exchange endoprosthetic reconstruction with tumour prosthesis for periprosthetic knee infection associated with segmental bone defects. Strateg. Trauma Limb Reconstr..

[16] Maupin J.J., Corning E., Steinmetz R.G., White J. (2019). Creating a dual articulating antibiotic spacer for management of an infected total femur prosthesis hemiarthroplasty. Arthroplasty Today.

